# Amygdala-Targeted Relief of Neuropathic Pain: Efficacy of Repetitive Transcranial Magnetic Stimulation in NLRP3 Pathway Suppression

**DOI:** 10.1007/s12035-024-04087-7

**Published:** 2024-04-04

**Authors:** Zhenhua Zhang, Zixin Hou, Mingming Han, Peng Guo, Kemin Chen, Jie Qin, Yuanzhang Tang, Fengrui Yang

**Affiliations:** 1 Department of Anesthesiology, Hunan University of Medicine General Hospital (The First People’s Hospital of Huaihua), No. 144, South Jinxi Road, Huaihua, 418000 Hunan Province P. R. China; 2 Department of Anesthesiology, The First Affiliated Hospital of University of South China, Hengyang, 421001 P. R. China; 3 Department of Anesthesiology, Division of Life Sciences and Medicine, The First Affiliated Hospital of USTC, University of Science and Technology of China, Hefei, 230036 Anhui P. R. China; 4 Department of Anesthesiology and Critical Care Medicine, The Johns Hopkins University School of Medicine, Baltimore, MD 21287 USA; 5 Department of Pain Management, Xuanwu Hospital, Capital Medical University, No. 45 Changchun Street Beijing, Beijing, 100053 P. R. China; 6 Board of Governors Regenerative Medicine Institute, Cedars-Sinai Medical Center, Los Angeles, CA 90048 USA

**Keywords:** Neuropathic pain, Repetitive Transcranial Magnetic Stimulation, Amygdala, Integrin αvβ3, P2 × 7R, NLRP3 Inflammatory Pathway

## Abstract

This study investigates the effectiveness of repetitive transcranial magnetic stimulation (rTMS) as a nonpharmacological approach to treating neuropathic pain (NP), a major challenge in clinical research. Conducted on male Sprague-Dawley rats with NP induced through chronic constriction injury of the sciatic nerve, the research assessed pain behaviors and the impact of rTMS on molecular interactions within the amygdala. Through a comprehensive analysis involving Mechanical Withdrawal Threshold (MWT), Thermal Withdrawal Latency (TWL), RNA transcriptome sequencing, RT-qPCR, Western blotting, immunofluorescence staining, and Co-Immunoprecipitation (Co-IP), the study focused on the expression and interaction of integrin αvβ3 and its receptor P2X7R. Findings reveal that rTMS significantly influences the expression of integrin αvβ3 in NP models, suggesting an inhibition of the NP-associated NLRP3 inflammatory pathway through the disruption of integrin αvβ3-P2X7R interactions. These outcomes highlight the potential of rTMS in alleviating NP by targeting molecular interactions within the amygdala, offering a promising therapeutic avenue for managing NP.

**Supplementary Information:**

The online version contains supplementary material available at 10.1007/s12035-024-04087-7.

## Introduction

Neuropathic pain (NP) is caused by direct or indirect damage to the nervous system and is a common symptom of many conditions that will result in impaired quality of life [1]. NP is common in the general population, with 7–10% of adults presenting with chronic NP [2]. Approximately 30% of patients with advanced cancer are affected by NP [3]. NP is a major clinical challenge, and although it has been extensively studied, its molecular mechanisms remain elusive. Although it has been shown that the Wnt/β-catenin pathway is involved in the persistent dorsal root ganglion compression-induced NP caused by chronic compression in a dorsal root ganglion model [4] and that clavulanic acid has a reducing effect on diabetic NP in rats [5], the detailed mechanisms underlying the occurrence and persistence of NP are unclear. Therefore, research into the mechanisms of NP development has become an important focus in the medical field.

Integrin αvβ3 is a heterodimeric adhesion protein that is highly expressed on endothelial cells and osteoclasts. Previous studies have shown that integrin αvβ3 can regulate tumor growth, metastasis, and size by acting on signaling pathways such as those of MAPK, FAK/AKT, ERK, and SRC/E2F1 [6–8]. Astrocytes release cytokines and participate in the regulation of neurons and synapses; thus, they affect the occurrence and maintenance of NP [9]. Furthermore, the expression of integrin αvβ3 and related proteins regulates the release of cytokines by astrocytes [10].

P2 × 7R is the most unique subtype of purinergic receptor, which can initiate a series of signaling pathways, such as pathways for the activation of the NALP3 inflammasome, the activation of the mitogen-activated protein kinase pathway, the enhancement of NF-K B-mediated transcription of inflammatory cytokines, and the mediation of the release of various inflammatory cytokines such as IL-1β, IL-6, IL-18, and TNF-α. Clinical studies have found that electroacupuncture can alleviate NP by inhibiting the expression of P2 × 7R, and downregulating the expression of P2 × 7R in the spinal cord can suppress NP [11]. In addition, regulating the main signaling pathway for the activation of the NLRP3 inflammasome can intervene in the treatment of related diseases [12]. Previous studies have demonstrated that the pyroptosis and neuroinflammation mediated by the P2 × 7R/NLRP3 signaling pathway can lead to cognitive impairment in a mouse model of migraine [13]. Modulating the P2 × 7R/STAT3/NF-κB65/NLRP3 signaling pathway can alleviate inflammatory responses and exert neuroprotective effects [14]. Furthermore, dexmedetomidine exerts a protective effect on ischemic brain injury by inhibiting the P2 × 7R/NLRP3/Caspase-1 signaling pathway [15].

Repetitive transcranial magnetic stimulation (rTMS) can modulate the plasticity of neurons in various neurological disorders, such as stroke, Parkinson’s disease, psychiatric disorders, and Alzheimer’s disease. The potential mechanisms underlying rTMS-induced neural recovery involve synaptic plasticity, neuronal death, neurogenesis, immune responses, and blood‒brain barrier disruption [16]. The literature includes reports that rTMS can significantly alleviate chronic NP [17], and treating the motor cortex (M1) with rTMS can be effective in the management of NP [18]. The therapeutic effect of rTMS is associated with a significant reduction in NP; thus, rTMS can be used for the treatment of central NP [19]. Moreover, high-frequency rTMS may alleviate NP by inhibiting the activity and proliferation of astrocytes ipsilateral to neurons of the spinal dorsal horn [20]. There have also been studies that have validated the stimulation frequency and pulse number identified in rTMS trials for treating NP, demonstrating that 2,000 pulses of rTMS provide better pain relief than other pulse numbers [21].

The amygdala is a region closely associated with pain, as reported in numerous studies [22, 23]. Glutamatergic neurons in the paraventricular nucleus (PVT)-amygdala circuit have been found to mediate persistent neuropathic pain [24]. Additionally, noradrenergic and serotonergic neurons in the amygdala-prefrontal cortex (PFC)-periaqueductal gray (PAG)-spinal cord pathway have been found to be involved in neuropathic pain [25]. However, the specific role of the amygdala in rTMS treatment of neuropathic pain has not been reported yet. Therefore, we chose the amygdala as the target brain region for this study.

In our study, we demonstrate the important role of neural activity in NP by exploring the amygdala, which may be associated with integrin αvβ3 activation of P2 × 7R, and reveal that integrin αvβ3 and P2 × 7R may be key pathways for rTMS of the NP. Our study provides a new research direction for the clinical exploration of rTMS for NP, validates the important role of integrin αvβ3 as a therapeutic target for NP, and demonstrates the important influence of P2 × 7R and its downstream NLRP3 inflammatory pathway in the therapeutic efficacy of rTMS, thus providing further evidence supporting the clinical use of rTMS for the treatment of related diseases. In addition, our study provides a new theoretical basis for uncovering the molecular mechanism of NP.

## Materials and Methods

### Establishment of NP Rat Model

One hundred and twenty 6-week-old male Wistar rats weighing 200 ± 20 g were purchased from BIOMARS (Beijing, China). All rats were housed in a specific pathogen-free environment with free access to food and water for 5 days of acclimatization experiments. Room temperature and humidity were maintained at 22 ± 0.5 °C and 50–55%, respectively.

NP rat model establishment: A spinal nerve ligation method was used to establish an NP model according to the available literature. Briefly, a rat was fixed in the prone position on a wooden surgical board and then anesthetized with isoflurane (R510-22, RWD, Shenzhen, China). Next, a longitudinal incision was made parallel to the spine between the left posterior superior iliac crest and the spine, measuring approximately 2 cm. The skin, fascia and muscles were then separated to expose the transverse process on the left side of the 6th lumbar vertebra, and the two parallel spinal nerves (L5 and L6 spinal nerves) were visible by severing the connection between the transverse process and the conus with stabbing forceps and separating the medial nerve. After ligating the medial nerve with a 6.0 silk ligature, the surgical wound was closed layer by layer with saline irrigation [26–28].

### Experimental Design

The random number table method was used to divide the mice into 12 groups (10 mice per group): the sham group (sham-operated group), NP group (NP model group), NP + pseudostimulation group (pseudostimulation group), NP + rTMS group (continuous rTMS at 0.5 Hz), NP + sh-NC group (model mice injected with interfering negative lentivirus), NP + sh-integrin αvβ3 group (model mice injected with interfering virus), NP + sh-integrin αvβ3 group (model mice injected with interfering integrin αvβ3 lentivirus), NP + rTMS + oe-NC group (model mice treated with continuous rTMS at 0.5 Hz and an injection of overexpressing negative lentivirus), NP + rTMS + oe-integrin αvβ3 group (model mice treated with continuous rTMS at 0.5 Hz and an injection of integrin αvβ3-overexpressing lentivirus), NP + sh-P2 × 7R group (model mice injected with P2 × 7R-interfering lentivirus), NP + rTMS + oe-NC + Control (model mice treated with continuous rTMS at 0.5 Hz and a coinjection of overexpressing negative lentivirus and PBS), NP + rTMS + oe-P2 × 7R + Control group (model mice treated with continuous rTMS at 0.5 Hz and a coinjection of P2 × 7R-overexpressing lentivirus and PBS), and NP + rTMS + oe-P2 × 7R + NLRP3 inhibitor group (model mice treated with continuous rTMS at 0.5 Hz and a coinjection of P2 × 7R-overexpressing lentivirus and an NLRP3 inhibitor). Our study was approved and agreed upon by our animal ethics committee.

### Open Field Test

The open field experiment evaluates the behavioral state of animals by assessing their psychological and exploratory characteristics in a new environment. The experiment is conducted in a separate room, ensuring a quiet setting. The experimental apparatus consists of a testing box measuring 40 cm × 40 cm × 50 cm, with the floor divided into equally-sized squares (4 × 4). A camera installed above the apparatus is adjusted to align with the box floor. The computer records the number of times a rat enters the center and the duration of stay in the center for 5 min. After each trial, the box floor is wiped with alcohol and the next experiment is conducted after the odor has dissipated [29].

### Forced Swimming Test

The forced swimming experiment assesses the effects of rTMS stimulation on animals by observing changes in their immobility time due to despair under unavoidable stressful conditions. In this experiment, rats are placed in a swimming tank with a height, diameter, and depth of 50 cm, 35 cm, and 35 cm respectively. The water temperature is maintained at 25 ± 1 °C and the swimming duration is 6 min. The immobility time is recorded during the last 4 min, during which the rats show no struggling behavior but only minimal limb movements to keep their heads above the water, appearing in a floating state [30].

### rTMS Therapy

After 15 days of NP induction, the treated group of rats received daily rTMS sessions for 8 consecutive days, with each session lasting 5 min and starting at 8:30 AM. The transcranial magnetic stimulation device was purchased from Shanghai Hanfei Medical Equipment Co., Ltd. The equipment generated pulses at a frequency of 0.5 Hz with a duty cycle of 1 ms and a magnetic field intensity of 200 millitesla (mT). During the stimulation process, the animals were restrained and wrapped in cloth. The coil was fixed to the head position using tape, and the butterfly coil was placed and secured in the same position as the active stimulation, while the animals in the sham stimulation group had the magnetic stimulator turned off during the procedure. The experimental protocol was based on previous reports [31, 32].

### Chronic Viral Infection

The lentiviral packaging system was constructed using LV5-GFP (a lentiviral gene overexpression vector, Shanghai Jikai Gene, China) and pSIH1-H1-copGFP (a lentiviral short hairpin RNA [shRNA] fluorescence expression vector, Shanghai Jikai Gene, China). The overexpression of integrin αvβ3 (oe-integrin αvβ3) and P2 × 7R (oe-P2 × 7R) was established, as was the overexpression of a negative control (oe-NC) and the silencing of integrin αvβ3 (sh-integrin αvβ3), P2 × 7R (sh-P2 × 7R), and negative control (sh-NC) using lentiviral vectors. First, primers were designed and synthesized based on the precursor sequences of integrin αvβ3 and P2 × 7R in the rat genome (sh-NC: CCGGCAACAAGATGAAGAGCACCAACTCGAGTTGGTGCTCTTCATCTTGTTGTTTTTG; sh-integrin αv: GACTGAGCTAATCTTGAGAAT; sh-integrin β3: CATTATGTTTACAGAGGACAA; sh-P2 × 7R: CCCGGCTACAACTTCAGATAT); sh-P2 × 7R: CCCGGCTACAACTTCAGATAT). The target fragments containing the integrin αvβ3 and P2 × 7R precursors were amplified by PCR using primers, and after enzymatic digestion, they were ligated into the vector. The adenoviral vectors carrying the integrin αv, integrin β3, and P2 × 7R precursors, along with the helper plasmids, were transfected into HEK293T cells (purchased from Nanjing Kebo Biotechnology Co., Ltd., Jiangsu, China) through intravenous injection. The supernatant was collected 48 h after cell culture and contained virus particles following filtration and centrifugation [33, 34]. Viral titers were determined. NP rats were infected with the virus by intrathecal injection every 3 days for a duration of 2 weeks. A virus dose of 1 × 10^8^ viral particles suspended in 20 μL of PBS was administered [33, 35, 36].

### Intramuscular Injection

After anesthesia with isoflurane (R510-22, RWD, Shenzhen, China), the rats were placed in a sitting position. The L5-6 interspinous space was chosen as the puncture site, and a microsyringe was slowly inserted into the gap. Following the occurrence of tail flicking in the rat, pressure was applied to the side of the rat’s neck over the jugular vein. The drug was administered slowly after the withdrawal of blood into the microsyringe, and the needle was spun out after the completion of administration [33].

### Mechanical Withdrawal Threshold and Thermal Withdrawal Latency Measurement

A series of Von Frey filaments (2, 4, 6, 8, 10, 15 g) (North Coast Medical, San Jose, CA, USA) were used to measure the mechanical withdrawal threshold (MWT) [37]. Each filament (ranging from 2.0 to 26.0 g) was applied to the plantar surface of each hind paw ten times. The testing started with a 2 g filament and gradually increased until the rat exhibited a response, retracting its paw from the surface of the testing apparatus. The plantar surfaces of the medial, ipsilateral, and contralateral hind paws were examined. Once a response was detected, lighter filaments were used in sequence to estimate the sensory threshold for each paw. The MWT was calculated using the following formula: 50% paw withdrawal threshold (g) = (10 ^ 255 [Xf + kδ])/10,000. The thermal withdrawal latency (TWL) of the plantar surface of the rat’s paw was measured using a paw plantar algesiometer (Tes7370, Ugo Basile, Comerio, Italy) [38]. The lateral aspect of the paw was exposed to a heating plate (50 °C), and the initial withdrawal latency and duration were recorded. Three heat stimulations were applied to each paw at 10-min intervals, and the average value was calculated [39, 40].

### RNA Extraction and Sequencing

Total RNA was isolated using TRIzol reagent (15,596,026, Invitrogen, Car, Cal, USA). rTMS-stimulated (0.5 Hz) and NP-group rat amygdala tissues were surgically obtained, and the concentration and purity of RNA samples were determined using a Nanodrop 2000 spectrophotometer (1011U, Nanodrop, USA) instrument. Purity. Total RNA samples meeting the following requirements were used for subsequent experiments: RNA integrity index (RIN) ≥ 7.0 and 28 S:18 S ratio ≥ 1.5.

Sequencing libraries were generated and sequenced by CapitalBio Technology (Beijing, China). A total of 5 μg RNA was used per sample. Briefly, ribosomal RNA (rRNA) was removed from total RNA using the Ribo-Zero™ Magnetic Kit (MRZE706, Epicenter Technologies, Madison, WI, USA). The NEB Next Ultra RNA Library Preparation Kit (#E7775, NEB, USA) was used for Illumina and to construct libraries for sequencing. The RNA was then fragmented into fragments of approximately 300 base pairs (bp) in length in NEB Next First Strand Synthesis Reaction Buffer (5×). First-strand cDNA was synthesized using reverse transcriptase primers and random primers, and second-strand cDNA was synthesized in second-strand synthesis reaction buffer in dUTP Mix (10×). end repair of cDNA fragments, including the addition of polyA tails and ligation of sequencing junctions. After ligation of the Illumina sequencing junction, the second strand of cDNA was digested using USER Enzyme (#M5508, NEB, USA) to construct strand-specific libraries. The library DNA was amplified, and the library DNA was purified and enriched by PCR. Libraries were then identified by Agilent 2100 and quantified using the KAPA Library Quantification Kit (KK4844, KAPA Biosystems, USA). Finally, paired-end sequencing was performed on a NextSeqCN500 (Illumina, USA) sequencer.

### Bioinformatics Analysis of Sequencing Data

The quality of the paired-end reads of the raw sequencing data was checked using FastQC software v0.11.8 (www.bioinformatics.babraham.ac.uk). Cutadapt software 1.18 (www.bioinformatics.babraham.ac.uk) was used for the preprocessing of raw data and the removal of Illumina sequencing junctions and poly(A) tail sequences. Reads with more than 5% N content were removed by Perl scripts. 70% of reads with more than 20 base pairs were extracted using FASTX Toolkit software 0.0.13 (http://hannonlab.cshl.edu/fastx_toolkit/). 70% of reads with more than 20 base pairs were extracted using BBMap software (https://sourceforge.net/projects/bbmap/) to repair double-ended sequences. Finally, the filtered fragments of high-quality reads were compared to the rat reference genome by HISAT2 software (0.7.12).

The mRNA-based read count was analyzed using the R language “edgeR” package for differential expression of mRNAs, setting |log2FC|>1 and P.value < 0.05 as the differentially expressed gene screening criteria. Gene Ontology (GO) and Kyoto Encyclopedia of Genes and Genomes (KEGG) enrichment analyses were performed using the “clusterProfiler” package.

### RT‒qPCR for the Relative Expression of Integrin αv, Integrinβ3 and P2 × 7R mRNA

RNA was extracted from tissues and cells to be tested using TRIzol reagent (15,596,026, Invitrogen, Car, Cal, USA). cDNA was reverse-transcribed from RNA according to the instructions of the PrimeScript RT reagent Kit (RR047A, Takara, Japan). cDNA synthesis was tested by a Fast SYBR Green PCR kit (Applied Biosystems) with an ABI PRISM 7300 RT‒PCR system (Applied Biosystems) for RT‒qPCR detection. The reaction system was as follows: SYBR Mix (9 μl), positive primer (0.5 μl), negative primer (0.5 μl), cDNA (2 μl), and RNase-free ddH_2_O (8 μl). Reaction conditions were as follows: 95 °C for 10 min, 95 °C for 15 s, and 60 °C for 1 min, 40 consecutive cycles. Three biological replicates were set up for each sample. GAPDH was used as an internal reference, and the relative expression at the mRNA level was analyzed using the 2^−ΔΔCt^ method: ΔΔCt = (average Ct value of target genes in the experimental group - average Ct value of housekeeping genes in the experimental group) - (average Ct value of target genes in the control group - average Ct value of housekeeping genes in the control group) [41]. The primer sequences are shown in Table S1.

### Immunofluorescence Staining and Immunohistochemical Staining

Rat amygdala tissue was fixed overnight in Bouin fixative, dehydrated, embedded in paraffin and sectioned longitudinally. The tissue paraffin sections were dewaxed in water, dehydrated in an alcohol gradient, washed in tap water for 2 min, soaked in 3% methanol/H_2_O_2_ for 20 min, and washed in distilled water for 2 min and 0.1 M PBS for 3 min. The sections were repaired in antigen repair solution in a water bath and cooled naturally. Normal goat blocking solution (C-0005, Shanghai Haoran Biotechnology Co., Ltd., Shanghai, China) was then added dropwise on the sections, which were left at room temperature for 20. The slides were shaken dry at room temperature for 20 min. Primary antibodies were added dropwise to the tissue slides, and the slides were incubated in the primary antibodies overnight at 4 °C (Tables S2), followed by 3 washes in 0.1 M PBS for 5 min/wash. Goat anti-rabbit IgG H&L (Alexa Fluor® 488) (ab150077, 1:2000, Abcam, Cambridge, UK) secondary antibodies were added dropwise to the tissue slides, and the slides were incubated in the secondary antibodies at 37 °C for 1 h. The sections were washed 3 times with PBST for 5 min each and then incubated for 5 min with a drop of DAPI on the slide while protected from light. Following four washes with PBST for 5 min each, the sections were blotted dry with absorbent paper and sealed with a blocking solution containing a fluorescent quencher.

The steps prior to incubation with the primary antibody were the same as those for immunofluorescence staining. Sections were incubated in primary antibody overnight at 4 °C, rewarmed to room temperature for 30 min, and then washed three times for 5 min/wash in 0.1 M PBS. Tissue pieces were titrated with goat anti-rabbit IgG (ab6785, 1:1000, Abcam, Cambridge, UK) secondary antibody, placed at 37 °C for 20 min, titrated with horseradish peroxidase Ltd., Beijing, China) at 37 °C for 20 min, DAB (ST033, Guangzhou Weijia Technology Co., Ltd., Guangzhou, China) was added dropwise to slides, which were then washed with water after color development. After incubation in hematoxylin (PT001, purchased from Shanghai Bogu Biotechnology Co.), the sections were washed with water; 1% ammonia was used for bluing, a certain concentration of gradient alcohol was used to dehydrate the sections, and xylene was used to clear the sections. Neutral resin was used to seal the sections. The sections were observed and photographed under a microscope. Five high magnification fields were randomly selected for each section, with 100 cells in each field. Each experiment was repeated three times.

### Gorky Staining

The Golgi staining procedure was performed in strict accordance with the instructions provided in the Golgi staining kit (HTKNS1125NH, Beijing Biolead Technology Development Co., Ltd., Beijing, China, http://www.bjbiolead.com/). Freshly dissected brains were immersed in solutions A and B, avoiding exposure to light, for a period of two weeks, followed by immersion in solution C at 4 C, also avoiding light, for two days. Solutions D and E were used as directed, and the tissues were then sliced using a microtome (SM2010R; Leica, Nussloch, Germany). Gradual dehydration was conducted with ethanol at concentrations of 100%, 95%, 75%, and 50% (2 min each for two cycles), followed by xylene clearing (5 min each for two cycles). The tissue sections were air-dried in a fume hood, mounted with neutral gum, and observed under an optical microscope. The number of neuronal dendritic spines in the amygdala was quantified, focusing on secondary or tertiary branch-like structures. Density measurement was based on the total length of dendrites in 1 mm3 of neural tissue, quantitatively analyzed across different tissue planes. The number of spines per micrometer of dendritic length was determined from 10 selected images per mouse obtained from digital photographs. The term “high magnification” refers to individual representative dendritic spine images that were enlarged [42].

### Western Blot Evaluation of Protein Expression in Rat Amygdala Tissue

After the completion of the physiological tests, the rats were sacrificed, and the tissue from the amygdala was extracted for Western blot evaluation of total protein expression. A 50 mg tissue sample was cut and added to Protein Lysis Solution (R0010, Solebro Technology Co., Ltd., Beijing, China) at a ratio of 150–250 μl of lysis solution per 20 mg of tissue and then homogenized at 1400 g until fully lysed. The supernatant was removed by centrifugation at 4000 × g for 15 min at 4 °C in an ice bath for 30 min. The protein concentration was determined according to the instructions in the BCA Protein Quantification Kit (23,225, Pierce, Rockford, IL, USA) and adjusted to 1 μg/μL. The processed proteins were added to the loading blanks, and 20 μg of sample was added per well. sodium dodecyl sulfate‒polyacrylamide gel electrophoresis (SDS‒PAGE) gels (10%, P1200, Solebro Technology Co., Ltd., Beijing, China) were used to separate the proteins by electrophoresis. Electrophoresis was first carried out at 8 v/cm and then at 15 v/cm after samples entered the separation gel. Electrophoresis was stopped when the sample was near the bottom of the separation gel. The protein samples were transferred to PVDF membranes (HVLP04700, Millipore, Bedford, MA, USA) by the semidry electrotransfer method and stained with Lichon Red (P0012, Beijing Solabond Technology Co., Ltd., Beijing, China) to observe the protein transfer. The membranes were washed twice with TBST, and the membranes were blocked with 5% skim milk powder at room temperature for 2 h. The membranes were then washed three times with TBST. The PVDF membranes were incubated with diluted primary antibody overnight at 4 °C (Tables S2) and washed 3 times with TBST for 10 min each. The membranes were incubated with HRP-labeled goat anti-rabbit IgG secondary antibody (S0001, Affinity) for 1 h, rinsed with TBST and placed on a clean glass plate. Medium amounts of Solution A and B of the ECL Fluorescence Detection Kit (Item BB-3501, Ameshame, UK) were mixed in a dark room, added dropwise to the membrane, and placed in a gel imager for exposure imaging and image capture. The ratio of the grayscale value of the target protein to the internal reference was used as the relative expression content of the protein.

### Co-IP Assay to Verify the Interaction of Integrin β3 with P2 × 7R

The amygdala tissue from rats was homogenized and lysed in a cell extraction buffer containing a protease inhibitor (1 mM EDTA, 150 mM NaCl, 20 mM Tris, pH 8.0, and 10% glycerol) obtained from Roche. The lysate was centrifuged at 4 °C and 4000 × g for 15 min, and the supernatant was collected. The protein concentration was determined using a BCA protein quantification assay kit (23,225, Pierce, Rockford, IL, USA), and the concentration was adjusted to 1 μg/μL. Thirty microliters of the supernatant was mixed with 30 μL of 2× SDS loading buffer as the input sample. The remaining supernatant was incubated with anti-P2 × 7R antibody or IgG (ab172730, Abcam, Cambridge, UK) at 4 °C with gentle rotation for 3 h. Protein A/G-Sepharose beads were added to the lysed cells and incubated overnight at 4 °C with gentle rotation. After washing, the immunocomplexes were boiled in SDS‒PAGE sample buffer for 5 min. The samples were separated by SDS‒PAGE, transferred onto nitrocellulose membranes, and blocked with 5% skim milk powder for 2 h. The membrane was then incubated overnight at 4 °C with primary antibodies against P2 × 7R or integrin β3, with β-actin as the loading control. After the membrane was washed with TBST, it was incubated at room temperature with secondary antibodies for 1 h. Following another round of washing with TBST, the membrane was visualized using the ECL detection system.

### Statistical Analysis

SPSS 21.0 (SPSS, Inc., Chicago, IL, USA) statistical software was used to analyze the data. Data are presented as the mean ± standard deviation. Data were first tested for conformity to a normal distribution, and those that conformed to a normal distribution were compared with the chi-square test or an unpaired t test for between-group comparisons, a one-way ANOVA for multiple group comparisons, the Tukey post hoc test or a two-way ANOVA for data at different time points or ANOVA for repeated measures data. *p* < 0.05 indicates statistically significant differences.

## Results

### rTMS Improves the Pain Phenotype and Abnormal Pathological Changes in the Amygdala of NP Rats

**Fig. 1:**
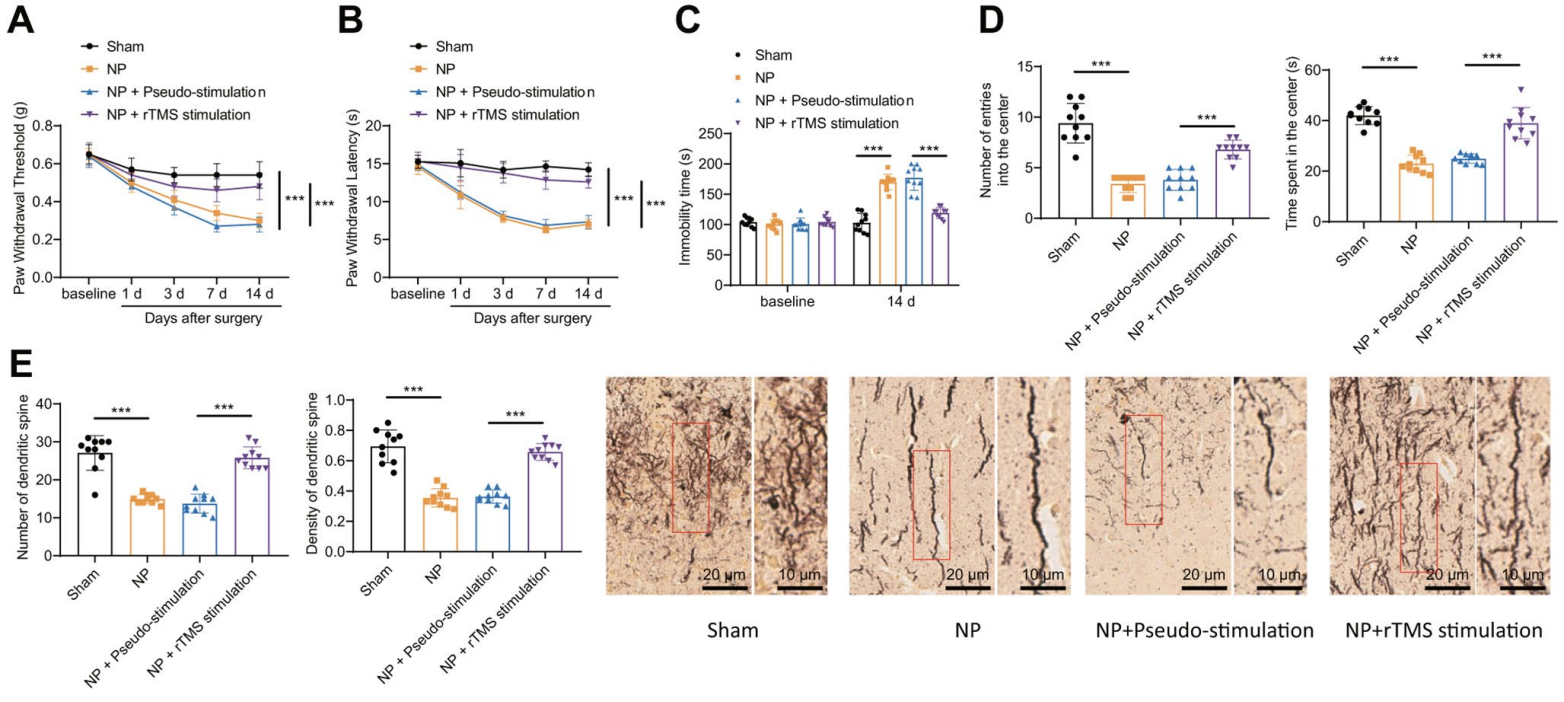
rTMS therapeutic effect on NP rats Note: **A**: comparison of mechanical pain thresholds in each group of rats; **B**: comparison of thermal pain thresholds in each group of rats; **C**: comparison of resting time of rats in water; **D**: comparison of the number of times rats entered the center and the time spent in the center in each group of rats; **E**: number and morphology of neurons in the amygdala dendritic spines in each group of rats detected by Golgi staining. * indicates a significant difference between groups (*P* < 0.05 for each group of 10 rats). The experimental results of the mouse groups were analyzed using a univariate approach. Pairwise comparisons between multiple groups were then performed using the Tukey post hoc test

To investigate the specific molecular mechanism of rTMS of the NP, we constructed an NP rat model and administered rTMS to NP rats. As shown in Fig. 1A-D, compared with the Sham group, NP rats developed nociceptive hyperalgesia, as evidenced by significantly lower mechanical and thermal pain thresholds, significantly increased resting time during forced swimming, and reduced number of center entries and time spent in the center during the null field test; these behavioral manifestations were reversed when NP rats were stimulated with rTMS. We examined the number and morphology of neuronal dendritic spines in the amygdala by Golgi staining and showed (Fig. 1E) that the number and density of neuronal dendritic spines in the amygdala were significantly reduced in NP rats compared to sham rats, while the number and density of neuronal dendritic spines in the amygdala were significantly increased in NP rats after rTMS.

These results indicate that rTMS can alleviate the abnormal number and morphology of neuronal dendritic spines in the amygdala of NP rats, thus alleviating the pain phenotype of NP rats.

### The Role of Amygdala Neural Activity in the NP may be Related to Integrin αvβ3

**Fig. 2:**
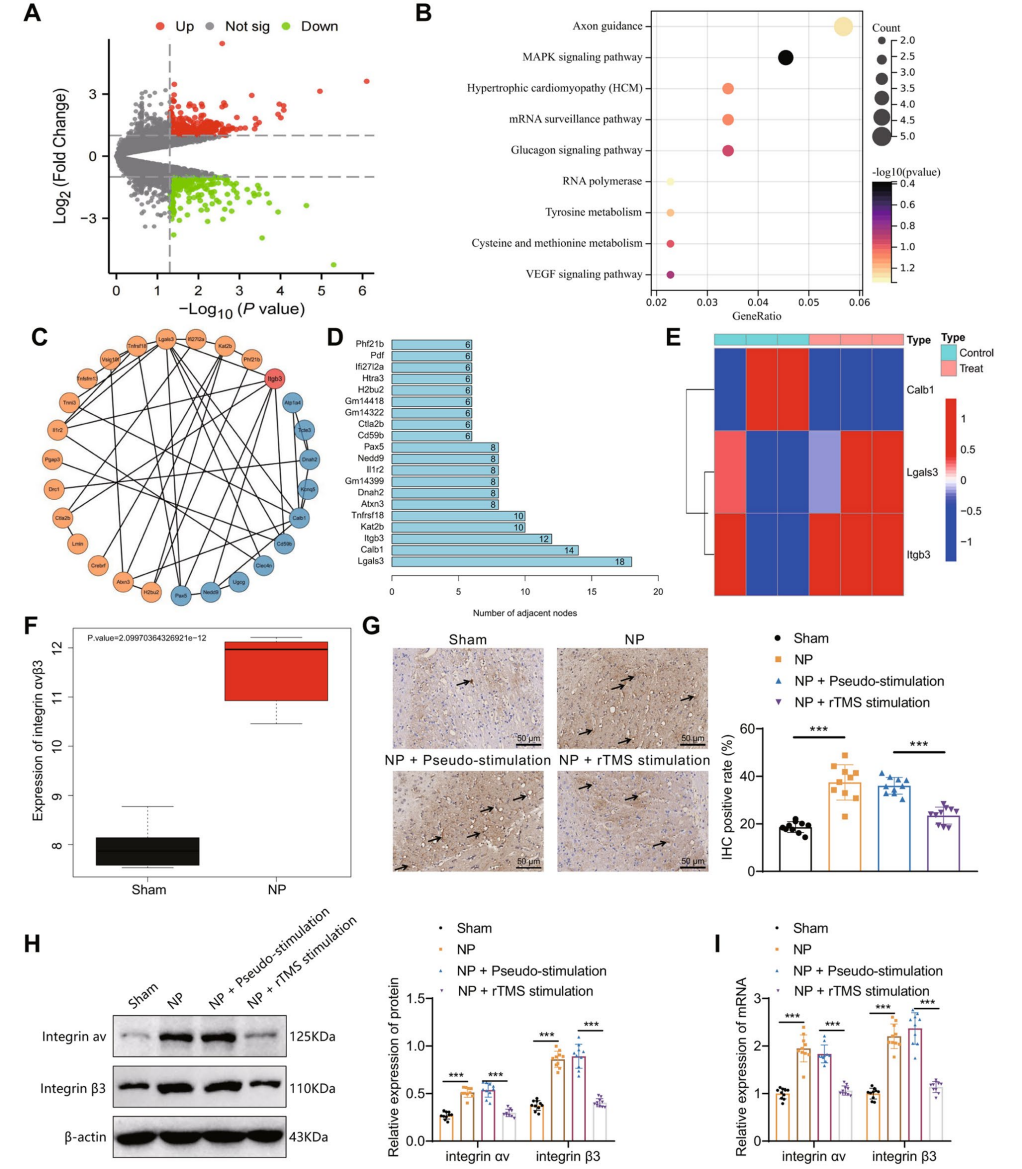
Neural activity in the amygdala in relation to integrin αvβ3 in NP Note: **A**: Expression volcano plot of differentially expressed genes between the NP group (*n* = 3) and rTMS-stimulated group (*n* = 3) rat amygdala tissue samples; **B**: KEGG enrichment analysis plot of differentially expressed genes, gene color scale blue to red indicates log2FC values of genes from negative to positive; **C**: PPI relationship network of integrin αvβ3 signaling pathway genes; **D**: The core gene adjacency node statistics of the gene interaction network are presented in a scatter plot, where the x-axis represents the adjacency node values and the y-axis represents the gene names. **E**: The heatmap reveals the differential expression of three candidate target genes in the sequencing data. The samples used in the analysis were from the amygdala tissues of rats in the rTMS group (Control, *N* = 3) and the NP group (Treat, *N* = 3). **F**: The boxplot displays the expression levels of integrin αvβ3 in the mRNA sequencing results. **G**: Representative images and statistical analysis of integrin αvβ3 immunohistochemistry in the amygdala tissues of each experimental group are shown. The scale bar represents 50 μm. (The arrows indicate the presence of integrin αvβ3-positive cells in the amygdala tissues of the rats.) **H**: Representative images and statistical analysis of integrin α and integrin vβ3 protein expression in the amygdala tissues of each experimental group are shown. **I**: The mRNA expression of integrin αv and integrin β3 in the amygdala tissues of each experimental group was evaluated using qPCR. The experimental results of the mouse groups were analyzed using a univariate approach. Pairwise comparisons between multiple groups were then performed using the Tukey post hoc test. * indicates a significant difference between the two groups (*P* < 0.05 for each group of 10 rats)

Chronic constriction injury of the sciatic nerve (sciatic nerve chronic constriction injury) was used to establish a rat model of NP. Surgical procedures were conducted to obtain amygdala tissue from three rats in the rTMS stimulation group (0.5 Hz) and the NP group for RNA transcriptome sequencing. After data quality control, mRNA identification was performed, followed by differential analysis of mRNA counts using the “edgeR” package in R programming language. A total of 219 significantly upregulated genes and 96 downregulated genes were identified (Fig. 2A).

To further identify key factors, we performed KEGG enrichment analysis on all differentially expressed genes mentioned above (Fig. 2B). The results showed that the differentially expressed genes were mainly involved in signaling pathways such as the MAPK signaling pathway and VEGF signaling pathway. We further selected the top 200 genes ranked by |log2FC| for interaction analysis. After removing free genes and genes with low relevance, we constructed a gene interaction network (Fig. 2C). The number of adjacent nodes for each gene in the network was calculated, and it was found that three genes had more than 10 adjacent nodes (LGALS3, CALB1, integrin αvβ3) (Fig. 2D). LGALS3 and integrin αvβ3 showed upregulated expression in the sequencing data, while CALB1 showed downregulated expression. Moreover, the differential expression of integrin αvβ3 was the most significant (Fig. 2E). Previous studies have shown the involvement of integrin αvβ3 in NP regulation [43], and the mRNA sequencing results indicated significantly higher expression of integrin αvβ3 in the amygdala tissues of rats in the NP group than in the control group (Fig. 2F). Therefore, we propose that amygdala neural activity might be associated with integrin αvβ3 in the NP.

Immunohistochemical staining revealed a significant increase in integrin αvβ3-positive cells in the amygdala tissue of NP rats compared to the Sham group. In contrast, the NP + rTMS group showed a significant decrease in integrin αvβ3-positive cells in the amygdala tissue compared to the NP + pseudostimulation group (Fig. 2G). Protein immunoblotting and RT-qPCR results indicated a significant increase in the protein and mRNA expression of integrin αv and integrin β3 in the amygdala tissue of NP rats (Fig. 2H-I).

The above results suggest that integrin αvβ3 is upregulated in the amygdala tissue of NP rats and that this effect can be reversed by rTMS treatment.

### rTMS may Alleviate Pain Phenotypes in NP Rats by Reducing Integrin αvβ3 Expression

**Fig. 3:**
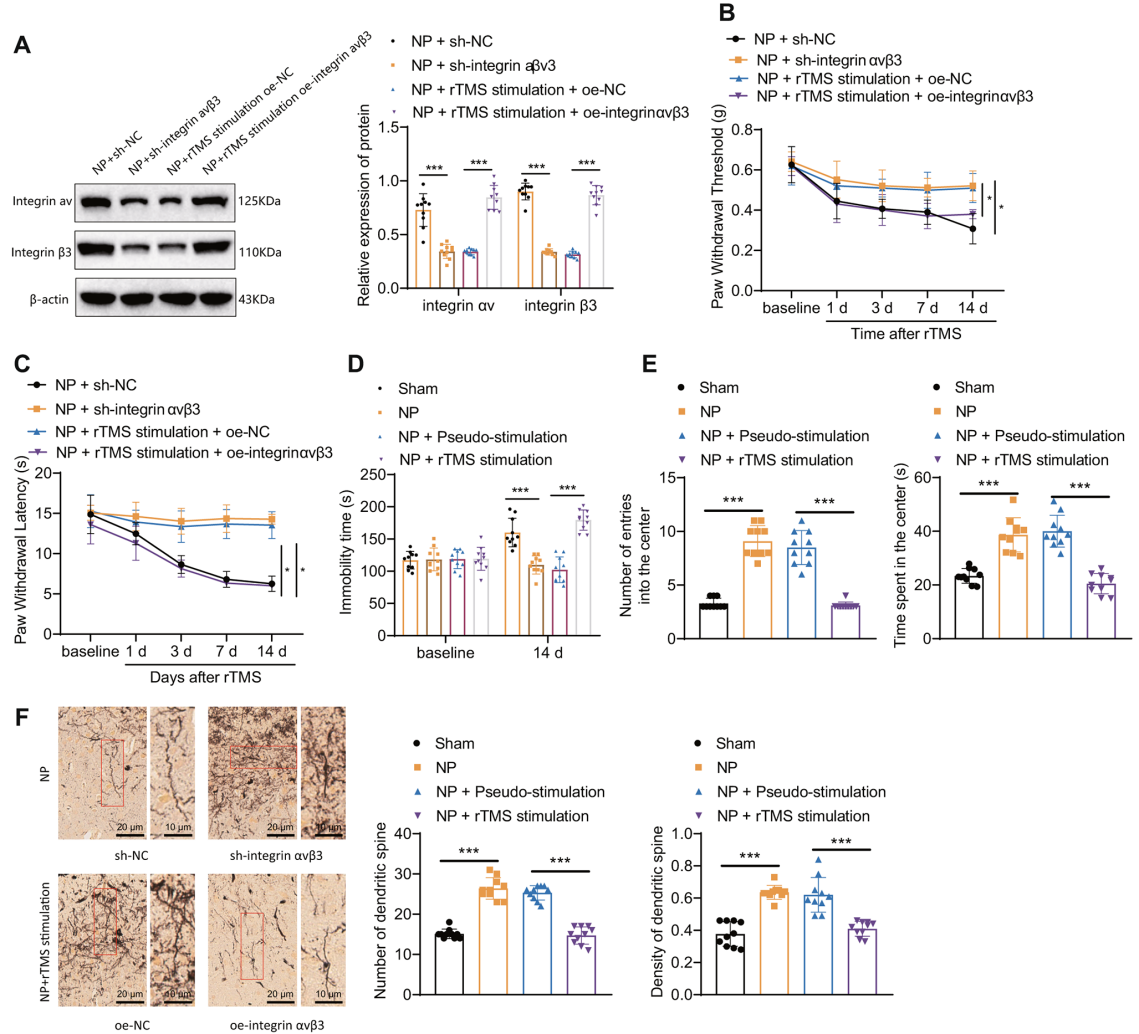
rTMS may alleviate pain in NP rats by reducing integrin αvβ3 expression Note: **A**: Western blot evaluation of integrin αv and integrin β3 protein expression levels in the amygdala tissue of each group; **B**: comparison of the mechanical pain thresholds of each group; **C**: comparison of the thermal pain thresholds of each group; **D**: comparison of the resting time of rats in water; **E**: comparison of the number of times rats entered the center and the time spent in the center of each group; **F**: Golgi staining method to determine the number and morphology of neuronal dendritic spines in the amygdala of each group. The experimental results of the mouse groups were analyzed using a univariate approach. Pairwise comparisons between multiple groups were then performed using the Tukey post hoc test. * indicates a significant difference between the two groups (*P* < 0.05 for each group of 10 rats)

To further investigate the effect of rTMS on pain characterization in NP rats by regulating integrin αvβ3 expression, we transfected NP rats with sh-integrin αvβ3 lentivirus or rTMS combined with oe-integrin αvβ3 lentivirus treatment. The expression of integrin αv and integrin β3 protein were significantly decreased in the amygdala of rats in the NP + sh-NC group compared with the NP + sh-integrin αvβ3 group, and integrin αv and integrin β3 protein was significantly increased in the amygdala of rats in the NP + rTMS + oe-integrin αvβ3 group compared with the NP + rTMS + oe-NC group (Fig. 3A).

As shown in Fig. 3B-E, compared with rats in the NP + sh-NC group, rats in the NP + sh-integrin αvβ3 group had significantly higher mechanical and thermal pain thresholds, significantly less resting time during forced swimming, and an increase in the number of times entering and staying in the center during the null field test. Compared with rats in the NP + rTMS + oe-NC group, rats in the NP + rTMS + oe-integrin αvβ3 group had significantly lower mechanical and thermal pain thresholds and significantly less resting time during forced swimming. The rats in the NP + rTMS + oeNC group had significantly lower mechanical and thermal pain thresholds, significantly increased resting time during forced swimming, and a decrease in the number of times entering and staying in the center during the null field test compared to rats in the NP + rTMS + oeNC group. The number and density of neuronal dendritic spines in the amygdala were measured by Golgi staining, and the results showed (Fig. 3F) that the number and density of neuronal dendritic spines in the amygdala were significantly increased in the NP + sh-integrin αvβ3 group compared to the NP + sh-NC group. Compared to the NP + rTMS + oe-NC group, the number and density of neuronal dendritic spines in the NP + rTMS + oe-NC group were significantly increased. The number and density of neuronal dendritic spines in the amygdala of rats in the rTMS + oe-integrin αvβ3 group were significantly reduced compared with those of rats in the NP + rTMS + oe-NC group.

These results suggest that rTMS may alleviate the abnormal number and morphology of neuronal dendritic spines in the amygdala of NP rats by reducing integrin αvβ3 expression, thereby alleviating the pain phenotype of NP rats.

### rTMS Inhibits the Interaction of Integrin αvβ3 with P2 × 7R in the Amygdala Tissue of NP Rats

**Fig. 4:**
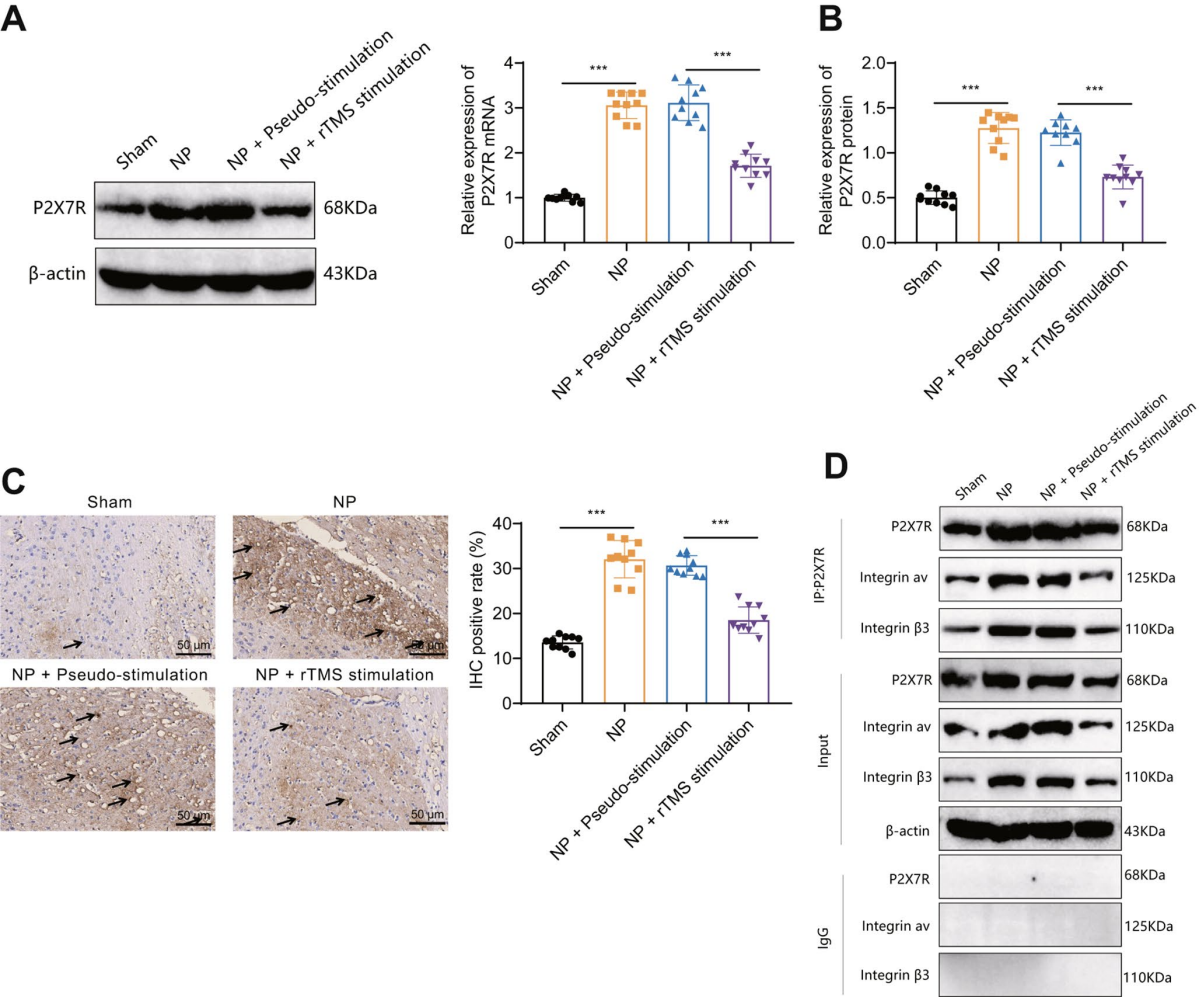
rTMS inhibits the interaction between integrin αvβ3 and P2 × 7R in the amygdala tissue of NP rats Note: **A**: Western blot to evaluate the expression levels of P2 × 7R in the basolateral amygdala tissue of rats at different periods; **B**: RT‒qPCR was used to determine the mRNA expression levels of P2 × 7R at different time intervals in the brain tissues of each group of rats; **C**: Immunofluorescence staining to detect P2 × 7R expression in the basolateral amygdala tissue of rats (arrows point to P2 × 7R-positive cells in the amygdala tissue of rats); **D**: Co-IP to evaluate the interaction between integrin αv and integrin β3 and P2 × 7R in tissue from the basolateral amygdala of rats from each group. The experimental results of the mouse groups were analyzed using a univariate approach. Pairwise comparisons between multiple groups were then performed using the Tukey post hoc test. * indicates a significant difference between the two groups (*P* < 0.05 for each group of 10 rats)

Integrin αvβ3 has been shown to be effective in alleviating anxiety through binding to P2 × 7R-acting receptors [44]. To further investigate the involvement of integrin αvβ3 in the pathogenesis of NP through the regulation of P2 × 7R, we utilized RT‒qPCR and Western blotting to assess the expression levels of P2 × 7R in the basolateral amygdala (BLA) tissues of rats in different groups. The results revealed a significant increase in the protein expression of P2 × 7R in the BLA tissues of NP rats compared to the Sham group. NP rats subjected to rTMS exhibited a significant decrease in P2 × 7R protein expression in BLA tissues when compared to NP rats receiving pseudostimulation (Fig. 4A). Moreover, the mRNA expression of P2 × 7R in the BLA tissues of NP rats showed a significant elevation compared to the Sham group, whereas NP rats receiving rTMS demonstrated a significant reduction in P2 × 7R mRNA expression in the BLA tissues in comparison to NP rats receiving pseudostimulation (Fig. 4B).

Immunohistochemical results also showed that the expression of P2 × 7R was significantly increased in the NP group; this effect was reversed by rTMS treatment (Fig. 4C). The interaction between integrin αv, integrin β3 and P2 × 7R was reduced in the amygdala tissue of the NP + rTMS-stimulation group compared with the Sham group (Fig. 4D).

The above results indicate that rTMS inhibits the interaction of integrin αvβ3 with P2 × 7R in the amygdala tissue of NP rats.

### rTMS Inhibits the NLRP3 Inflammatory Signaling Pathway by Regulating P2 × 7R Expression in NP Rats

**Fig. 5:**
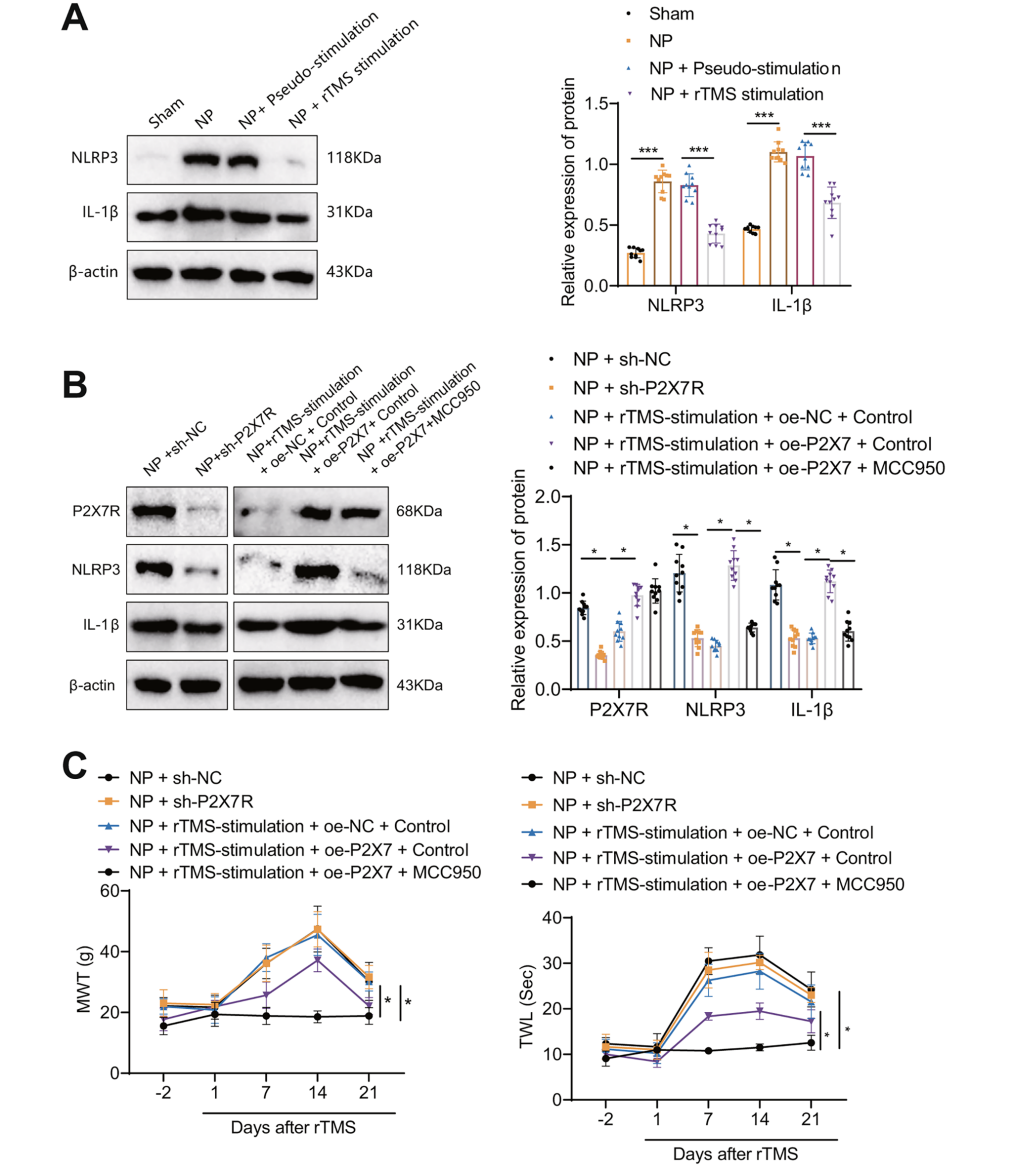
rTMS inhibits the NLRP3 inflammatory signaling pathway by regulating P2 × 7R expression to treat NP in model rats Note: **A**: Western blot experiments to evaluate the expression of NLRP3 and IL-1β in basolateral amygdala tissue samples from each group of rats at different periods; **B**: Western blot to evaluate the expression levels of P2 × 7R, NLRP3 and IL-1β in basolateral amygdala tissue samples from each group of rats; **C**: MWT and TWL levels of NP rats after different treatments (0 indicates preinjection). The experimental results of the mouse groups were analyzed using a univariate approach. Pairwise comparisons between multiple groups were then performed using the Tukey post hoc test. * indicates a significant difference between the two groups (*P* < 0.05 for each group of 10 rats)

It has been shown that the NLRP3 inflammatory pathway is activated in NP and thus involved in the development of NP and that this pathway is directly related to P2 × 7R [13, 45]. To further explore whether rTMS inhibits the NLRP3 inflammatory signaling pathway by regulating P2 × 7R expression and thus treats NP in model rats, we first examined the expression of NLRP3 inflammatory signaling pathway-related proteins (NLRP3, IL-1β) in tissue from the GLA of rats in each group using Western blotting, and the results showed that compared with the Sham group, NP rats showed significantly increased expression of NLRP3 and IL-1β, while the expression of NLRP3 and IL-1β were significantly decreased in the amygdala tissues of NP + rTMS-stimulation rats compared to NP + Pseudostimulation rats. This finding serves as a reminder that rTMS treatment has the potential to reverse the alterations induced by NLRP3 and its downstream inflammatory signal, IL-1β, in NP (Fig. 5A).

Subsequently, we treated the NP group rats with sh-P2 × 7R lentivirus transfection, rTMS combined with oe-P2 × 7R lentivirus treatment or rTMS combined with oe-P2 × 7R lentivirus and an NLRP3 inflammatory signaling pathway antagonist (MCC950 at 50 mg/kg intraperitoneally). Western blot results showed that compared with the NP + sh- NC group, the expression of P2 × 7R, NLRP3 and IL-1β was significantly reduced in the amygdala tissue of rats in the NP + sh-P2 × 7R group compared with the NP + rTMS-stimulation + oe-NC + Control group. Compared with the NP + rTMS-stimulation + oe-P2 × 7 + Control group, the expression of P2 × 7R, NLRP3 and IL-1β was significantly reduced in the amygdala tissue of rats in the NP + rTMS-stimulation + oe-P2 × 7 + Control group. The expression of P2 × 7R, NLRP3 and IL-1β was significantly increased in the amygdala tissue of rats in the NP + rTMS-stimulation + oe-P2 × 7 + Control group compared to rats in the NP + rTMS-stimulation + oe-P2 × 7 + NLRP3 inhibitor group. The expression of P2 × 7R, NLRP3 and IL-1β was unchanged in the amygdala tissue of rats in the NP + rTMS-stimulation + oe-P2 × 7 + Control group. NLRP3 and IL-1β expression was significantly reduced in the amygdala tissue of rats in the NP + rTMS-stimulation + oe-P2 × 7 + Control group. This observation indicates that the beneficial effects of rTMS may be counteracted by high expression of P2 × 7, while partial restoration can be achieved by blocking NLRP3 (Fig. 5B). The MWT and TWL were measured at each time point, and the results showed that compared with rats in the NP + sh-NC group, the rats in the NP + sh-P2 × 7R group showed a reduced pain phenotype. Compared with the NP + rTMS-stimulation + oe-NC + Control group, the rats in the NP + rTMS-stimulation + oe-P2 × 7 + Control group showed an increased pain phenotype. Compared with the NP + rTMS-stimulation + oe-NC + Control group, the rats in the NP + rTMS-stimulation + oe-P2 × 7 + Control group showed an increase in the pain phenotype. Rats in the NP + rTMS-stimulation + oe-P2 × 7 + NLRP3 inhibitor group showed a decrease in the pain phenotype compared to rats in the NP + rTMS-stimulation + oe-P2 × 7 + MCC950 group (Fig. 5C).

These results suggest that rTMS can inhibit the NLRP3 inflammatory pathway by regulating P2 × 7R expression and thus treat NP in model rats.

## Discussion

rTMS uses pulsed magnetic fields to act on the cerebral cortex, which alters the membrane potential of nerve cells, causing them to generate induction currents, thereby affecting brain metabolism and neurophysiological activity, promoting the reconstruction of central neural networks, regulating the secretion levels of various transmitters and functional brain networks, and thus treating neuropsychiatric disorders [46]. In recent years, the use of rTMS has been widely reported for the treatment of NP, and clinical data have demonstrated that rTMS can effectively improve the symptoms of NP in patients. Some studies have been conducted to optimize the parameters of rTMS to obtain better symptom relief [47]. Several studies have reported the potential of rTMS in improving the pathological state of the amygdala and treating severe depression [48]. The amygdala plays a crucial role in the development of NP. However, there is no clear research on whether the therapeutic effect of rTMS on NP is achieved through its impact on amygdala activity.

Currently, most research on integrin αvβ3 is focused on cancer and neural activity. Inflammatory reactions can induce the migration and response of astrocytes mediated by αvβ3 integrin, and astrocytes play a significant role in regulating the process of NP [10]. Additionally, integrin αvβ3 plays a vital role in the precise modulation of neural network function [49]. It has been reported that integrin αvβ3 is involved in the development of depression by regulating the expression of the central brain neuron protein SERT [50]. Furthermore, integrin αvβ3 can interact with the P2 × 7R receptor, activating downstream inflammatory signaling pathways, which are crucial for the occurrence and development of NP [44]. However, the mechanistic role of integrin αvβ3 in the NP process has not been extensively studied.

Our study found that rTMS can improve pain characteristics and pathological abnormalities in the amygdala of NP rats. Furthermore, through transcriptomic sequencing, we identified a potential association between amygdala neural activity and integrin αvβ3 in the context of NP. This suggests that integrin αvβ3 plays a crucial role in the development and occurrence of NP and can serve as a therapeutic target for intervention. In our co-IP experiment, we observed a significant enhancement of the interaction between integrin β3 and P2 × 7R in the amygdala of NP rats, indicating that their interaction may be one of the pathways involved in NP development. By examining the downstream signaling pathways of P2 × 7R, we discovered that the activation of the NLRP3 inflammatory signaling pathway by P2 × 7R is an intrinsic mechanism underlying NP. This finding highlights that an excessive inflammatory response triggered by P2 × 7R downstream activation, particularly through the NLRP3 signaling pathway, is a direct cause of NP. Targeting NLRP3 or employing anti-inflammatory therapies could serve as novel treatment strategies for NP.

We also found that the activation of the NLRP3 inflammatory pathway by P2 × 7R may be intrinsic to the development of NP, and the results suggest that the inflammatory overreaction activated downstream of P2 × 7R is the direct cause of NP, with the NLRP3 signaling pathway being the main contributor. The involvement of P2 × 7R in the headache process has already been reported, as P2 × 7R is involved in the migraine process by activating NLRP3 and thus releasing IL-1β during migraine development [51]. Additional articles have also demonstrated that P2 × 7R/NLRP3 is a key factor in migraine-related cognitive impairment and may be a potential therapeutic target for alleviating migraine cognitive impairment [13].

In addition, in vivo animal studies further demonstrated that rTMS can treat NP in model rats by inhibiting the interaction between integrin αvβ3 and P2 × 7R. This result demonstrates the underlying mechanism of rTMS treatment of NP and provides the first evidence that the blocking of the interaction of integrin αvβ3 with P2 × 7R can be used as a separate therapeutic target for NP. Integrin αvβ3 has been reported to activate P2 × 7R by triggering the release of ATP through binding to Syndecan-4 [52], but the direct interaction of integrin αvβ3 with P2 × 7R through protein‒protein interactions has not been reported. Furthermore, this study has not yet examined the dosage of rTMS used to treat NP in rats in relation to the dosage used in humans.

## Conclusions

**Fig. 6:**
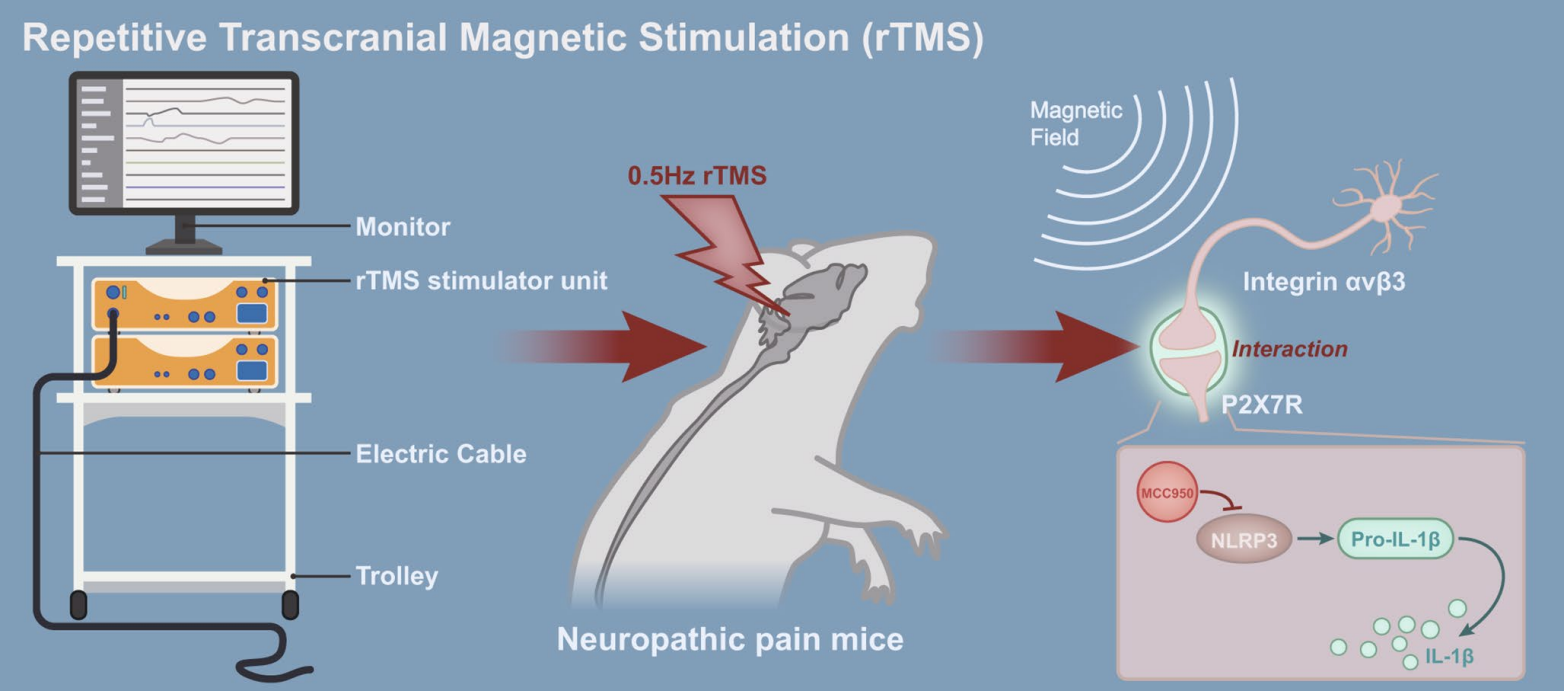
Schematic representation of the molecular mechanism by which repetitive transcranial magnetic stimulation relieves neuropathic pain by blocking integrin α_v_β_3_ from interacting with P2 × 7R in the amygdala and inhibiting the NLRP3 inflammatory signaling pathway

In summary, we conclude that rTMS can alleviate NP by blocking the interaction between integrin αvβ3 and P2 × 7R in the amygdala and inhibiting the NLRP3 inflammatory pathway (Fig. 6). Our study explored the activation of the NLRP3 inflammatory pathway by P2 × 7R during the development of NP as a direct mechanism of action and the inhibition of the interaction of integrin αvβ3 with P2 × 7R by rTMS as an intrinsic mechanism for its treatment of NP. However, our study also has the following limitations. First, our study only validated and explored the interaction between integrin αvβ3 and P2 × 7R, and our current findings demonstrate the relevance of integrin αvβ3 and P2 × 7R in the treatment of NP by rTMS but not the direct interaction between integrin αvβ3 and P2 × 7R. Integrin αvβ 3 may be correlated with P2 × 7R by a variety of mechanisms, possibly transcription-dependent regulation. It is also possible that the complex is formed through auxin rather than the direct binding of integrin αvβ3 to P2 × 7R.

## Electronic Supplementary Material

Below is the link to the electronic supplementary material.

Supplementary Material 1

## Data Availability

The data that support the findings of this study are available in the manuscript and supplementary materials.

## Abbreviations

MWT: Mechanical withdrawal threshold
NP: Neuropathic pain
rTMS: Repetitive transcranial magnetic stimulation
