# Supplementary material for: Amygdala-Targeted Relief of Neuropathic Pain: Efficacy of Repetitive Transcranial Magnetic Stimulation in NLRP3 Pathway Suppression
Source: Mol Neurobiol. 2024 Apr 4;61(11):8904–20. doi: 10.1007/s12035-024-04087-7 (PMC11496354; doi:10.1007/s12035-024-04087-7)
Supplement: Supplementary file 1 — Supplementary Material 1 [file 12035_2024_4087_MOESM1_ESM.docx]

**Table S1. RT-qPCR primer sequences**

| Gene | Sequences |
| --- | --- |
| integrin αv | Forward: 5'-ATCTGTGAGGTCGAAACAGGA -3' |
|  | Reverse: 5'-TGGAGCATACTCAACAGTCTTTG-3' |
| integrin β3 | Forward: 5'-TTCAATGCCACCTGCCTCA-3' |
|  | Reverse: 5'-TGAAGCTCACCGTGTCTCCAA-3' |
| P2X7R | Forward: 5'-GCTGCTTGGGAAAAGTCTGC-3' |
|  | Reverse: 5'-TGGCACCAATCTGGGCTG-3' |
| GAPDH | Forward: 5'-ACAGCAACAGGGTGGTGGAC-3' |
|  | Reverse: 5'-TGAGGGTGCAGCGAACTT-3' |

**Table S2. Primary Antibody Product Information**

| Name | Cat. | Dilution ratio | Country |
| --- | --- | --- | --- |
| Rabbit integrin αv antibody | ab179475 | 1：500/125 kda | abcam/USA |
| Rabbit integrin β3 antibody | ab119992 | 1：500/110 kda | abcam/USA |
| Rabbit anti-P2X7R antibody | PA5-28020 | 1: 1000/69 kda | ThermoFisher/USA |
| Rabbit anti-NLRP3 antibody | MA5-32255 | 1: 1000/118 kda | ThermoFisher/USA |
| Mouse anti-IL-1β antibody | sc-52012 | 1: 500/30 kda | Santa Cruz Biotechnology/USA |
| Rabbit anti-GAPDH antibody | Ab181602 | 1: 1000/36 kda | Abcam/UK |
